# Piperidinols That Show Anti-Tubercular Activity as Inhibitors of Arylamine *N*-Acetyltransferase: An Essential Enzyme for Mycobacterial Survival Inside Macrophages

**DOI:** 10.1371/journal.pone.0052790

**Published:** 2012-12-28

**Authors:** Areej Abuhammad, Elizabeth Fullam, Edward D. Lowe, David Staunton, Akane Kawamura, Isaac M. Westwood, Sanjib Bhakta, Alun Christopher Garner, David L. Wilson, Peter T. Seden, Stephen G. Davies, Angela J. Russell, Elspeth F. Garman, Edith Sim

**Affiliations:** 1 Department of Pharmacology, University of Oxford, Oxford, United Kingdom; 2 Faculty of Pharmacy, University of Jordan, Amman, Jordan; 3 Department of Biochemistry, University of Oxford, Oxford, United Kingdom; 4 Department of Chemistry, University of Oxford, Oxford, United Kingdom; 5 Faculty of Science, Engineering and Computing Kingston University, Kingston, United Kingdom; University of Delhi, India

## Abstract

Latent *M. tuberculosis* infection presents one of the major obstacles in the global eradication of tuberculosis (TB). Cholesterol plays a critical role in the persistence of *M. tuberculosis* within the macrophage during latent infection. Catabolism of cholesterol contributes to the pool of propionyl-CoA, a precursor that is incorporated into cell-wall lipids. Arylamine *N*-acetyltransferase (NAT) is encoded within a gene cluster that is involved in the cholesterol sterol-ring degradation and is essential for intracellular survival. The ability of the NAT from *M. tuberculosis* (TBNAT) to utilise propionyl-CoA links it to the cholesterol-catabolism pathway. Deleting the *nat* gene or inhibiting the NAT enzyme prevents intracellular survival and results in depletion of cell-wall lipids. TBNAT has been investigated as a potential target for TB therapies. From a previous high-throughput screen, 3-benzoyl-4-phenyl-1-methylpiperidinol was identified as a selective inhibitor of prokaryotic NAT that exhibited antimycobacterial activity. The compound resulted in time-dependent irreversible inhibition of the NAT activity when tested against NAT from *M. marinum* (MMNAT). To further evaluate the antimycobacterial activity and the NAT inhibition of this compound, four piperidinol analogues were tested. All five compounds exert potent antimycobacterial activity against *M. tuberculosis* with MIC values of 2.3–16.9 µM. Treatment of the MMNAT enzyme with this set of inhibitors resulted in an irreversible time-dependent inhibition of NAT activity. Here we investigate the mechanism of NAT inhibition by studying protein-ligand interactions using mass spectrometry in combination with enzyme analysis and structure determination. We propose a covalent mechanism of NAT inhibition that involves the formation of a reactive intermediate and selective cysteine residue modification. These piperidinols present a unique class of antimycobacterial compounds that have a novel mode of action different from known anti-tubercular drugs.

## Introduction

Tuberculosis (TB) remains the leading cause of death by bacterial infection [1]. According to WHO reports, latent infection represents the major pool of worldwide TB cases, making the treatment of latent TB an important strategy towards eradicating the disease [2]. Persistence of *Mycobacterium tuberculosis* (*M. tuberculosis*) within the host's macrophages is the hallmark of latent infection [3].

The unique lipids of the mycobacteria cell wall have been shown to contribute to the persistence of mycobacteria within the macrophage and to play an important role in the virulence and pathogenicity of *M. tuberculosis* [4], [5]. Cholesterol has been shown to play an important role in the entry of mycobacteria into macrophages [6]. Furthermore, *M. tuberculosis* is capable of using cholesterol as a carbon source inside the macrophage. The catabolism of cholesterol affects the propionate pool in mycobacteria and augments the production of virulence lipids [7]–[9]. Propionyl-CoA (Pr-CoA) is converted to methylmalonyl-CoA (Mm-CoA), which is considered to be the building block of multimethyl-branched mycolic acids such as Phthiocerol Dimycocerosate (PDIM) [8]. Several gene clusters that were shown to be involved in cholesterol degradation are also essential for mycobacterium survival inside the macrophage [10]–[12].

**Figure 1:**
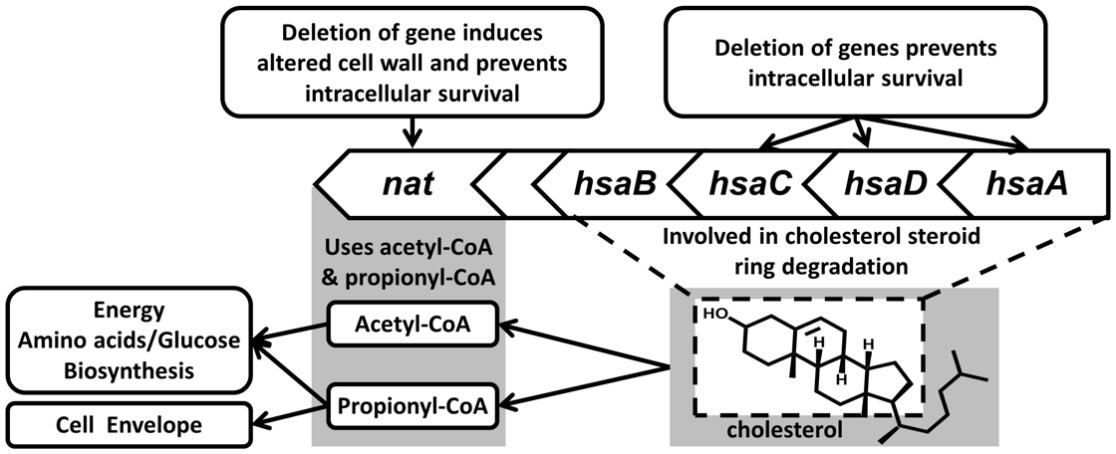
The gene cluster that encodes for the *nat* gene in *M. tuberculosis* and *M. bovis* BCG and its relation to cholesterol catabolism. The accession numbers, detailed at http://genolist.pasteur.fr/TubercuList/, for these genes in *M. tuberculosis* H37Rv are as follows: Rv3570c (*hsaA*), Rv3569c (*hsaD*), Rv3568c (*hsaC*), Rv3567c (*hsaB*), Rv3566A (possible pseudogene) and Rv3566c (*nat*). The gene cluster is virtually identical in *M. tuberculosis* and *M. bovis* BCG.

The catabolism of the sterol nucleus of cholesterol in *M. tuberculosis* involves the action of the *hsaADCB* products of a gene cluster which includes *nat* (Figure 1) [13], [14], the gene encoding for arylamine *N*-acteyltransferase (NAT). NAT utilises Pr-CoA in addition to acetyl-CoA (Ac-CoA) as an acyl donor, both of which are products of degradation of the alkyl moiety of cholesterol [15], [16]. Both whole genome [17] and candidate gene approaches [18], [19] have shown the importance of this gene cluster in the intracellular survival of mycobacteria.

NAT is a cytosolic enzyme that is found in *M. tuberculosis* and many other organisms [20]. This enzyme catalyses the transfer of an acyl group, usually an acetyl, to an arylamine substrate using a conserved cysteine residue by a Ping-Pong bi-bi mechanism [21]. The *nat* genes from *M. tuberculosis* and *M. bovis* Bacillus Calmette–Guérin (BCG) are identical and are encoded in virtually identical gene clusters in both organisms (Figure 1).

Deleting the *nat* gene from *M. bovis* BCG resulted in delayed growth and caused morphological changes of the BCG bacilli. Moreover, the Δ*nat* mutant severely lacked mycolic acids and virulence-lipid content (PDIM and the cord factor). These effects were overcome when the mutant strain was complemented with the target gene [19]. Chemical inhibition of the NAT activity within mycobacteria resulted in similar changes in morphology, cell-wall lipids and intracellular survival to those observed upon deleting the gene [22]. Furthermore, the chemically treated strains showed high sensitivity to gentamicin and hygromycin, which have weak activity against mycobacteria [19]. This enzyme is thus an attractive therapeutic target in the search for new anti-tubercular agents.

Despite the near-ubiquitous occurrence of the NAT enzyme, mycobacterial NATs appear to have distinguishing features from the eukaryotic enzymes [23]. Structural studies on the CoA bound forms of both Human NAT2-CoA (HNAT2-CoA, PDB code 2PFR) [24] and *M. marinum* NAT (MMNAT-CoA, PDB code 2VFC) [23], showed distinct binding sites for CoA in these two enzymes [25]. Interestingly, potent micromolar inhibitors of human NAT1, which have been investigated as a marker for breast cancer, did not exhibit any inhibition of mycobacterial NATs [26]. NAT inhibitors that are selectively toxic to mycobacteria, therefore, would remove any potential human toxicity caused by inhibition of the human NAT enzymes.

The search for novel drugs that can shorten the treatment course for TB has become pressing in the light of the shortcomings of the current therapy and the emergence of extensively-drug resistant (XDR) strains [27], [28]. New compounds with a variety of mechanisms of action are being developed and are in the preclinical and clinical phase [29], [30]. However, none of the current investigational compounds specifically targets cholesterol catabolism in mycobacteria or products of the gene cluster encoding NAT. Therefore, the development of novel inhibitors targeting these enzymes would provide new therapeutic options for the treatment of latent and XDR TB.

**Figure 2:**
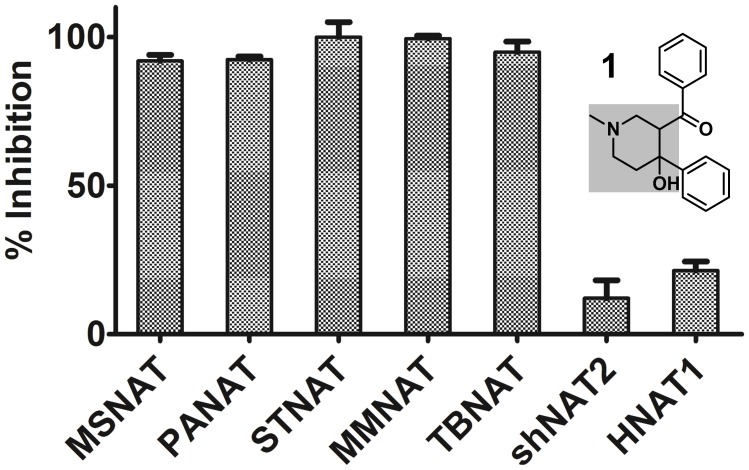
Specificity of compound 1 for prokaryotic NAT enzymes. Compound **1** was tested at 30 µM against pure recombinant NAT enzymes from *M. smegmatis* (MSNAT), *P. aeruginosa* (PANAT), *S. typhimurium* (STNAT), MMNAT and TBNAT, and also against two eukaryotic enzymes, hamster NAT2 (shNAT2) and human NAT1. The results are shown as the mean ± S.D. of triplicate determinations of the percentage inhibition of hydrolysis of Ac-CoA in the presence of 5-aminosalicylic acid (5ASA) and against TBNAT using hydralazine as a substrate. The inhibition is represented as a percentage compared to an uninhibited control from triplicate measurements. The structure of compound **1** is shown and the piperidinol nucleus is highlighted by the shaded area.

In a previous study, we have identified 3-benzoyl-4-phenyl-1-methylpiperidinol (compound **1**, Figure 2) by high-throughput screen (HTS) methods using pure recombinant NAT enzymes [22], [31]. In this study, the mechanism of NAT inhibition by this class using a selected panel of piperidinol analogues is investigated. A novel mechanism of NAT inhibition by the piperidinols is proposed. This class of inhibitors constitutes an attractive starting point for further drug development efforts against TB.

## Results and Discussion

### Evaluation of compounds for NAT inhibition and antimycobacterial activity

Compound **1**, a piperidinol derivative (Figure 2), was identified through high throughput screening against a panel of multiple pure recombinant NATs including mycobacterial, bacterial and eukaryotic isoenzymes [22], [31]. In order to confirm selectivity, the compound was re-evaluated against an extended panel of enzymes (Figure 2). Compound **1** shows high selectivity for bacterial and mycobacterial NATs over the eukaryotic enzymes, thereby satisfying the strict selection criteria considered in the original screening programme [22], [31]. The compound was in fact reported over 50 years ago for its antimycobacterial activity with a minimum inhibitory concentration (MIC) against *M. tuberculosis* of less than 5 µg/mL (∼17 µM) [32]. To explore the antimycobacterial potential and the NAT inhibition of the piperidinol class, four analogues (**2–5**; Table S1) with different aryl and *N*-functionality patterns were evaluated for their inhibitory activity against MMNAT and TBNAT, as shown in Table 1. All five compounds showed potent inhibition of both TBNAT and MMNAT (Table 1).

**Table 1:** The inhibitory activity of compound 1 and its analogues.^a^

Code	TBNAT		MMNAT				MIC (µM)^b^	
	% Inhibition	IC_50_ (µM)	% Inhibition	IC_50_ (µM)	k_obs_ (10^−3^ min^−1^)	t_1/2_ (min)	*M. bovis* BCG	*M. tuberculosis*
**1**	101±1	7.7±0.9	105±1	1.3±0.0	9±2	81.5	21.3–42.3	3.4–16.9
**2**	98±1	1.6±0.1	103±2	0.16±0.01	110±2	6.3	17.3–34.3	2.7–13.7
**3**	72±60	4.4±0.1	103±1	ND	573±18	1.2	17.3–34.4	2.8–13.8
**4**	67±4	1.1±0.3	100±2	2.7±0.4	586±115	1.2	14.8–29.3	2.3–11.7
**5**	101±2	1.2±0.1	101±2	0.14±0.02	34±2	20.4	21.3–42.3	3.4–16.9

a The NAT activity was measured by the NAT-inhibition assay using 150 µM HLZ and 120 µM Ac-CoA as substrates. The level of enzyme inhibition was measured in the presence of 50 µM inhibitor and compared to the un-inhibited control. The antimycobacterial activity against *M. bovis* BCG and *M. tuberculosis* were determined. Inhibition curves were obtained by non-linear fitting of the % inhibition and the inhibitor concentration (µM) using the Log(inhibitor) vs. response module of GraphPad Prism 5.0. The time-dependent assay *k_obs_* values were obtained from the slope of the semilogarithmic plots of the residual activity vs incubation time at 11.9 µM, except for **3** (5.9 µM). The results are presented as the mean ± S.D. of triplicate measurements at 24°C. t_1/2_ is the apparent inactivation half-life calculated from *k_obs_* (t_1/2_ = 0.693/k_obs_). ND is not determined.

b See Methods for further experimental details.

The compounds were also assessed for *in vitro* anti-mycobacterial activity against *M. bovis* BCG and the *M. tuberculosis* H37Rv (Table 1). All the tested compounds showed promising antimycobacterial activity against *M. tuberculosis* H37Rv with a MIC below 17 µM (Table 1). The compounds were also tested *in vitro* for their cytotoxic effect on RAW 264.7 cells, and no cytotoxicity was observed at up to 150 µM inhibitor concentration. Cytotoxicity results from tests with the same inhbitors in the U937 human cell line gave similar data.

### Irreversible NAT inhibition by the piperidinols

**Figure 3:**
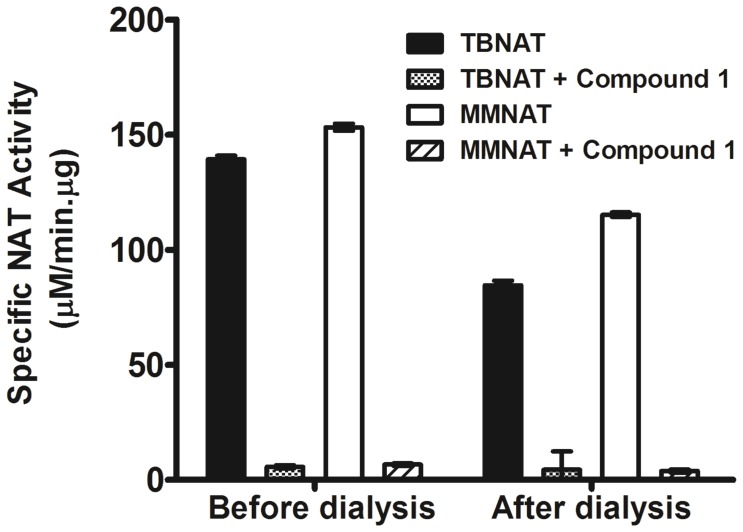
Reversibility of the inhibition of TBNAT and MMNAT by compound 1. Each enzyme (MMNAT, TBNAT, 0.07 mM, 50 µL) was preincubated either alone or with 15-fold molar excess 1 at 24°C for 1 h. Each sample was then dialysed against 1 L fresh assay buffer (20 mM Tris-HCl pH 8) at 4°C for 16 h. The enzyme activities of the samples were measured before dialysis and then measured after dialysis by measuring the rate of Ac-CoA hydrolysis in the presence of HLZ as described in Methods. The mean ± S.D. of three measurements of the activity is shown. Loss of enzyme activity upon dialysis is likely to be due to the oxidation of the active site sulfhydryl group, especially since dialysis was performed in the absence of dithiothreitol.

It was noted in the studies of inhibition of enzyme activity that the piperidinol compounds exhibited a time-dependent inhibition, which markedly increased with the time of incubation of NAT with the inhibitor (Figure S1). This type of inhibition is usually observed as a result of tight binding of the inhibitor to the enzyme, irrespective of whether this binding involves the formation of a covalent adduct with reactive residues in the protein [33]. Extensive dialysis of the enzyme after incubation with compound **1** was completely ineffective in restoring the activity of the enzyme, supporting the proposal of tight irreversible binding of **1** to the NAT enzyme (Figure 3). These data showed the same pattern for both MMNAT and TBNAT.

**Figure 4:**
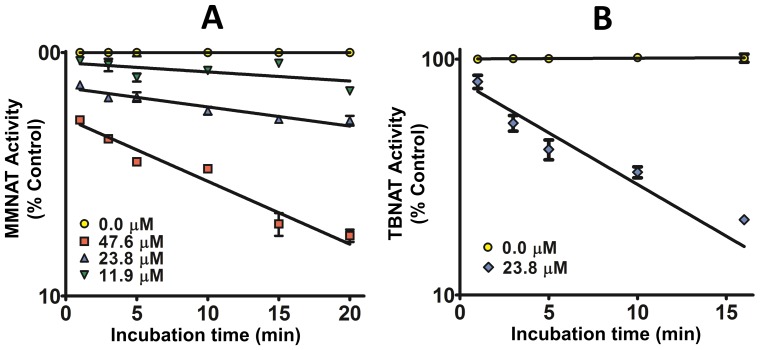
The time-dependent inhibition of the MMNAT and TBNAT by the piperidinols. Semi-logarithmic plots showing the time-dependent inactivation of (A) MMNAT by various concentrations of **1** and (B) TBNAT by compound **3** at 23.8 µM. The enzyme activity was measured using the protocol described in Figure S1. The results are presented as the mean ± S.D. of triplicate measurements. The residual activity is shown as a percentage of a control prepared as described in Figure S1. The data were fitted against the incubation-time using the Semilog line (X is linear, Y is Log) module of GraphPad Prism 5.0. The slope of each line is equivalent to (−k_obs_/2.303) at each inhibitor concentration. The error bars are within the symbols.

The time course of the inhibition of NAT by the piperidinols was evaluated according to the Kitz and Wilson model [34]. Irreversible inhibition, progressive with time, was measured for the piperidinol inhibitors (**1**–**5**) by the protocol described in Figure S2, and the values of the apparent first order constant (*k_obs_*) were determined (Table 1). Dilution rather than dialysis was used in this protocol due to the likely loss of activity upon dialysis (Figure 3). The piperidinols exhibited similar inhibition against TBNAT although higher concentrations of the inhibitor were required compared to those which inhibited MMNAT. Examples of the curves obtained for enzyme activity against incubation time upon inhibition of MMNAT and TBNAT by the piperidinols are shown in Figure 4.

### Covalent adduct formation as the mechanism of NAT inhibition

**Figure 5:**
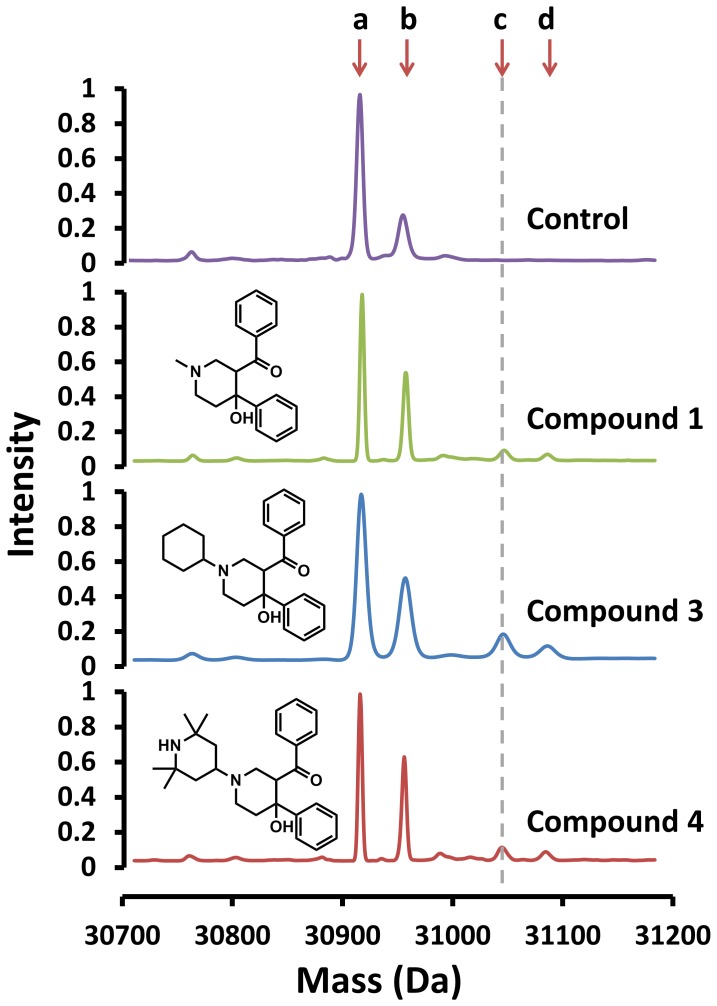
The ESI mass spectrum of MMNAT in the presence of 1, 3 and 4. MMNAT was mixed with an equimolar sample (1∶1 ratio) of each inhibitor (50 µM) in 20 mM Tris-HCl, pH 8, and 5% (v/v) DMSO, and the ESI-MS was performed after 30 min of incubation. The masses correspond to each peak according to MMNAT with compound **3** chromatogram are: a = 30915 (Δm = 0 Da), b = 30955.5 (Δm = 40 Da), c = 31046.5 (Δm = 131.5 Da), and d = 31087.5 Da (Δm = 172.2 Da). The mass corresponding to the addition of a 132 Da-fragment is marked with a dashed line. Δm of +40 Da is likely to correspond to a potassium ion (38 Da). A mass spectrum of the protein in the absence of any inhibitor is shown as control in the top panel.

**Figure 6:**
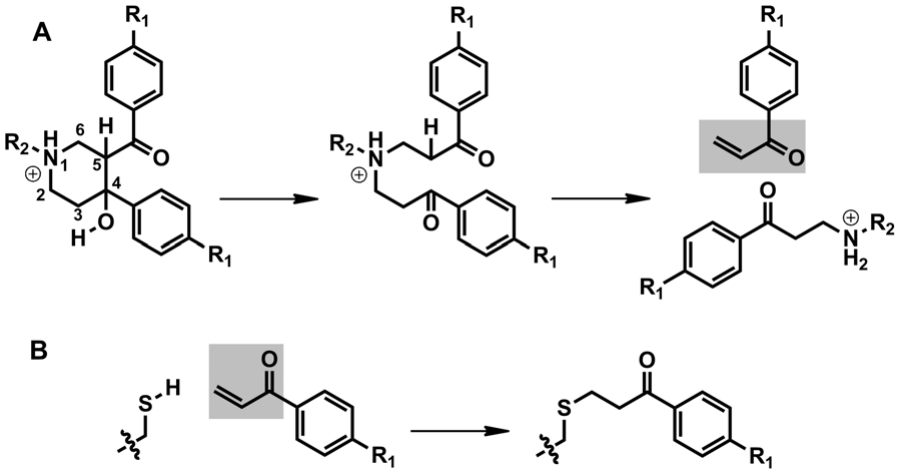
The chemical transformation of 1 to the corresponding phenyl vinyl ketone (PVK) and the subsequent modification of a thiol containing residue by the PVK. (A) A proposed pathway of the formation of bis-Mannich bases from the rigid cyclic piperidinol. The bis-Mannich base can undergo a β-elimination of the amino group forming a reactive phenyl vinyl ketone (PVK). (B) The PVK reaction with thiols resulted in the addition of a 3-phenyl-3-oxopropyl moiety (POP) (when R_1_ is H) or a 3-(4-chlorophenyl)-3-oxopropyl moiety (when R_1_ is Cl). The expected Δm values of the added fragments are +132.07 Da and +166 Da, respectively. The shaded areas highlight the Michael acceptor moiety. Since the PVK binding species is transient, the second order rate constant cannot be determined without major assumptions being made.

The ability of the piperidinols to form a covalent adduct with MMNAT was initially investigated by mass spectroscopic (MS) analysis of the protein-ligand complex at a molar ratio of 1∶1 using compounds **1–5** (Table 2, Figure 5). The mass differences (Δm) between the enzyme-inhibitor complexes and the enzyme alone are shown in Table 2 (upper section). A single additional peak which corresponds to a new species of higher molecular mass was observed for each of the protein-inhibitor complexes. These results confirm covalent adduct formation with each of the different piperidinols tested. Interestingly, all compounds with unsubstituted aryl groups (**1**, **3**, **4** and **5**) resulted in a protein adduct with an increase of the average molecular mass of 132 Da regardless of the *N*-substituents in these compounds (Table 2 and Figure 5). Compound **2** (the p-chloroaryl derivative of **1**) showed a mass of 164 Da compared with the other inhibitors (132 Da): however, this mass difference (i.e. 164 Da) from the native is approximately equivalent to an additional chlorine atom (35.5 Da) added to the 132 Da-fragment. These findings support the postulate that all inhibitors inactivate the enzyme by a general mechanism, which involves the formation of a reactive aryl compound that can react covalently with the enzyme. A proposed mechanism for the formation of the reactive intermediate is illustrated in Figure 6. The change in molecular mass expected from the addition of a 3-phenyl-3-oxopropyl moiety to the protein (C_9_H_8_O) is 132.07 Da, whilst the addition of a 3-(4-chlorophenyl)-3-oxopropyl moiety (C_9_H_7_ClO) is 166.03 Da (Figure 6A). These values are in agreement with the mass difference observed upon incubating the enzyme with an equimolar amount of each piperidinol (Table 2 and Figure 5). Furthermore, treatment of MMNAT and TBNAT with phenyl vinyl ketone (PVK) resulted in a mass difference of 132, in agreement with the proposed mechanism of activation. PVK showed 100% inhibition of the NAT activity within 10 minutes even at less than 1 µM concentration level.

**Table 2:** Mass spectrometric analysis results for MMNAT and TBNAT after incubation with the piperidinol inhibitors.

Sample	Deconvoluted mass (Da)	Δm (Da)	ΔDm/m_pvk_	Molar ratio (Inhibior/protein)	Cys
**Enzyme-to-inhibitor ratio of 1∶1**					
MMNAT-**1**	31047.6	132.6	0.9	1	3
MMNAT-**2**	31085.3	164.3	1	1	3
MMNAT-**3**	31046.5	131.5	1	1	3
MMNAT-**4**	31045.5	130.5	1	1	3
MMNAT-**5**	31050.6	129.7	1	1	3
MMNAT-PVK	31056.0	132	1	1	3
**Enzyme-to-inhibitor ratio of 1∶15**					
MMNAT-**1**	31185.9	255.9	1.9	2	3
	31062.6	132.6	1	1	3
	31324.1	394.1	3	3	3
MMNAT-**2**	31424.1	494.1	3	3	3
	31254.5	324.5	2	2	3
	31089.3	159.3	1	1	3
TBNAT-**1**	31450.9	134.1	1	1	2
	31583.4	266.6	2	2	2

For the ESI-MS studies, samples of each enzyme (0.07 mM in 20 mM Tris-HCl, pH 8.0 and 5–10% (v/v) DMSO) were incubated with the different inhibitors at an enzyme-to-inhibitor molar ratio of either 1∶1 or 1∶15 at 24°C for 30–60 min. Confidence intervals are ±8–12 Da. Samples of each enzyme alone (in the same buffer) were analysed in the same way as the controls and used to calculate the mass difference upon incubation with the inhibitor (Δm values). m_pvk_ is the mass of the expected phenyl-oxopropyl fragments of 132 Da for **1**, **3**, **4** and **5**, or 166 Da for **2** and identifies the number of modifications. The Molar ratio refers to the proposed number of inhibition species bound per protein molecule. Cys refers to the number of cysteine residues in the sequence. See Methods for further experimental details.

The activity of the acyclic bis-Mannich base **5** supported the hypothesis that the action of the piperidinols was mediated by conversion to the corresponding bis-Mannich base. Despite the high activity of compound **5**, the fact that it has been reported to exhibit greater toxicity compared to the cyclic piperidinol [35] made it less favoured for further investigation as an antimycobacterial compound.

**Figure 7:**
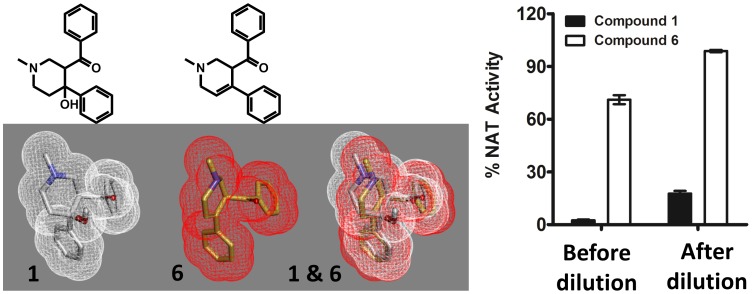
A comparison of the 3D-shape of compounds 1 and 6 and their inhibition activity. (A) The 3D-shape of compounds **1** and **6** are shown in a mesh view of the Van der Waals surface. Overlapping 3D-shapes of **1** (in white) and **6** are also shown. Energy minimisation of compounds **1** and **6** was performed using Grade (http://grade.globalphasing.org). The structure of **6** is shown. (B) The activity of MMNAT in the presence of 50 µM compound **1** or **6**. The activity of MMNAT was measured after incubation with 50 µM of each inhibitor for 20 min before and after a 200-fold dilution. The NAT activity was measured by the NAT-inhibition assay using 150 µM of HLZ and 120 µM Ac-CoA. The percentage of enzyme activity was measured in the presence of 50 µM inhibitor and compared to the un-inhibited control. The results are presented as the mean ± S.D. from triplicate measurements at 24°C.

Elimination of the hydroxyl group from compound **1** (compound **6**) resulted in 30% inhibition of the MMNAT at 50 µM inhibitor concentration (Figure 7). However, the inhibition of the enzyme with compound **6** was reversible, unlike the situation with compound **1** (Figure 7). Compound **6** has a similar 3-dimensional-shape to that of compound **1** (Figure 7) but lacks the hydroxyl group. Therefore, it is not expected to undergo the activation mechanism described in Figure 6.

In contrast, when MMNAT was incubated with a 15-fold molar excess of piperidinols **1** and **2**, the formation of multiple protein adducts was observed after MS analysis (Table 2, lower section). Compound **1** and MMNAT formed three distinct adducts, the mass of each of which matches the mono, di or tri derivatives of the 132 Da-fragment (Table 2; lower section). For compound **2**, three adducts were also observed corresponding to the mass of mono, di or tri derivatives of the 166 Da-fragment. The increase in molecular mass is depicted in Table 2 (lower section). MMNAT has three cysteine residues, and these results can be explained by the reaction of each of the piperidinols with each of the free sulfhydryl groups in MMNAT. Whilst this does not prove that the reaction has occurred with cysteine residues in MMNAT at 15∶1 molar ratio of piperidinol to MMNAT, it is indicative that this is very likely to be the case.

To investigate further the reaction of compound **1** with cysteine, a sample of the amino acid alone was reacted with the compound under conditions similar to those used in the protein experiment for Electrospray ionisation mass spectrometry (ESI-MS) analysis in parallel with untreated samples of both cysteine and compound **1**. The samples were treated with 6-aminoquinolyl-n-hydroxysuccinimidyl carbamate after reaction with the piperidinol and prior to liquid chromatography (LC) and MS analysis to facilitate separation and identification.

**Figure 8:**
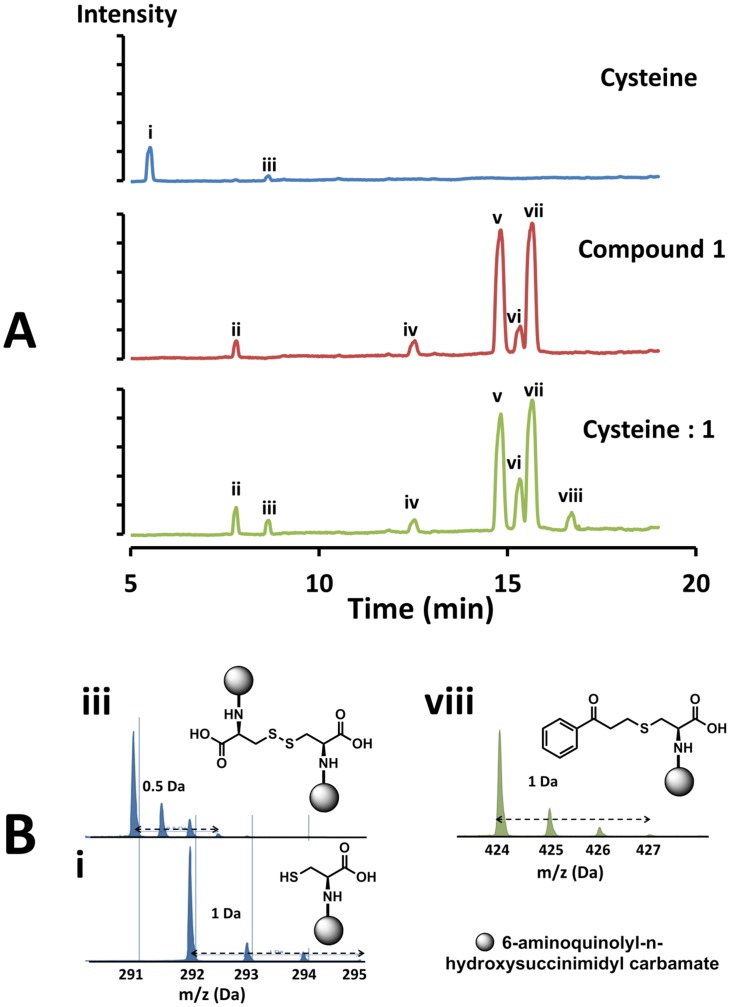
LC/MS analysis of the reaction of compound 1 with free cysteine. (A) The total ion current chromatogram (from liquid chromatography LC) of 100 µM cysteine, 100 µM compound **1** and 100 µM cysteine: **1** (1∶1 mixture) in 20 mM MOPS buffer, pH 8 after 16 h incubation at 24°C. All samples were treated with 6-aminoquinolyl-n-hydroxysuccinimidyl carbamate before analysis. (B) The ESI-MS spectra of fractions collected from the peaks in the chromatogram (in A) corresponding to i: cysteine (m/z = 291.9 Da), iii: cystine (m/z = 290.9) and viii: the product of the reaction of cysteine with **1** (m/z = 423.9 Da). The chemical structures of the compounds corresponding to each peak are shown. The round symbol represents the aminoquinolyl carbamate moiety.

The chromatogram of cysteine alone solution showed a major peak corresponding to the mass of the aminoquinolyl carbamate derivative of cysteine (m/z = 291 Da, peak i Figure 8) and a small peak corresponding to cystine, the disulphide dimer of cysteine (m/z = 290.9, peak iii Figure 8). When cysteine was incubated with compound **1**, the peak corresponding to cysteine (peak i, m/z = 291 Da) was absent from the chromatogram and an increase in the cystine peak (iii, Figure 8) was observed. In addition, there was a unique peak in the reaction of cysteine with compound **1**. This peak (viii in Figure 8) had a molecular mass of 423.9 Da. The difference between the mass of cysteine and that of the new entity (peak viii, Figure 8) was 132 Da, which is in agreement with the molecular mass difference observed upon the reaction of compound **1** with MMNAT. When compound **1** was treated alone in the same way, it gave multiple peaks, which were present in the same relative amounts as in the chromatogram obtained from the mixture of cysteine and compound **1**. The formation of an adduct with piperidinol compounds has been reported in the alkylation of cellular glutathione in human T cells [35]. The mechanism proposed for the formation of the resultant reactive PVK is similar to that shown in Figure 6B, proceeding through hydrolysis to the corresponding bis-Mannich base, followed by a β-elimination reaction of the secondary amine.

These results show that the piperidinols interact with cysteine. The observations suggest that in the presence of a 15-fold molar excess of inhibitor, the modification occurred indiscriminately with all three cysteine residues in the MMNAT sequence. However, when the molar ratio is 1∶1, only one inhibitor molecule is bound. Therefore, we propose that in the presence of an equimolar amount of the inhibitor, the active site cysteine Cys70 is the residue which is modified. The occurrence of Cys70 within a Cys-His-Asp triad affords activation of the cysteine sulfhydryl group [36]. However, the accessibility of compound **1** to the active site cysteine also appears to contribute to the modification, since compound **1** did not show significant inhibition of the eukaryotic NATs despite the presence of cysteine within the same active triad (Figure 2). Mycobacterial NAT enzymes have a CoA binding pocket which is distinct from that of eukaryotic enzymes [23].

In order to carry out MS analysis, the piperidinol was reacted with MMNAT in the native state, but prior to the MS measurements, acetonitrile denaturation was performed. Thus, in the presence of excess inhibitor, the excess piperidinol is likely to react with the cysteine residues exposed by the acetonitrile treatment and subsequent denaturation process.

The treatment of TBNAT with 15-fold excess of compound **1** resulted in two species the mass of each of which corresponds to the mass of mono and di derivatives of the 132 Da-fragment (Table 2). There are two cysteine residues in TBNAT, which further supports the interpretation of a reaction with the cysteine residues exposed during denaturation in the presence of excess piperidinol. The cysteine residues following denaturation become equivalent to those in glutathione and cysteine itself.

It is also interesting to note that the free cysteine can to react with these compounds, although it does require a long incubation time. This demonstrates that activation of these compounds to form reactive PVK in the presence of the enzyme is compatible with reaction through the active site cysteine.

### Structural studies on MMNAT-inhibitor complexes

To validate the observations obtained using MS, the 3D-structure of MMNAT was determined in complex with compound **1**, the parent piperidinol identified from the HTS.

**Figure 9:**
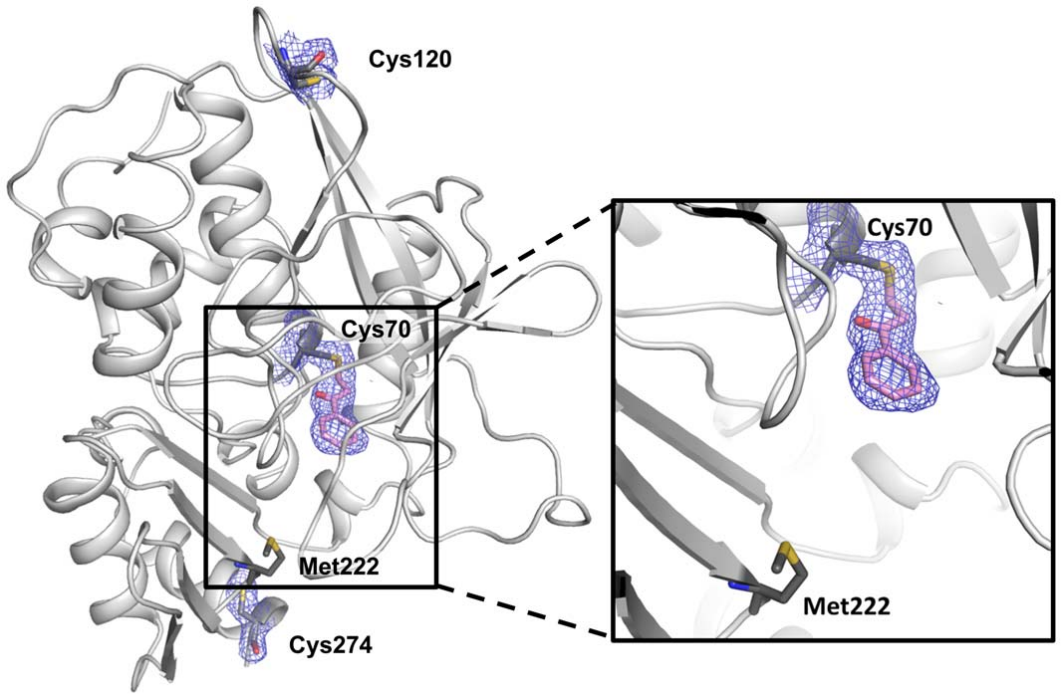
The active site electron density observed in the MMNAT-POP complex. The crystal structure of MMNAT after reaction with compound **1** showed excess electron density connected to Cys70, into which a 3-phenyl-3-oxopropyl (POP) modification was modelled with full occupancy. All three cysteine residues in the MMNAT structure and the covalent modification (in pink) are shown with the electron density shown using blue 2Fo–Fc electron density contoured at 1 σ. This observation is compatible with the MS data, since the excess inhibitor was washed out prior to crystallisation and the native state of the protein was preserved throughout the structure determination process. The figures were prepared using PyMOL [67].

The crystallographic structure determination was performed by incubating MMNAT with **1** in solution (15∶1 inhibitor to protein ratio in the native form) and then to crystallise it (co-crystallisation). According to the proposed mechanism of inhibition, this method is expected to allow time for the ligand to mature into the covalent modification of the active site cysteine with the 3-phenyl-3-oxopropyl moiety (POP). The crystals diffracted to 2.7 Å and data were processed as described in Methods and in Table S2. Upon refinement of the co-crystallised complex MMNAT-POP, a continuous excess electron density connected to the active site Cys70 was observed, consistent with the proposed 3-phenyl-3-oxopropyl modification (Figure 9). No electron density was observed in proximity to any of the other cysteine residues (Cys120 and Cys274; Figure 9) or any other residues, confirming the selectivity of the inhibitors to the active site cysteine. The binding site of the fragment which was observed attached to the active site cysteine was accommodated by the fragment predicted from the MS studies using a 1∶1 ratio of compound **1** to MMNAT. These data are entirely compatible with the MS data obtained using the same compound to MMNAT ratio, since the protein remains in the native state throughout the crystallisation and excess inhibitor was removed by buffer exchange prior to crystallisation as described in Methods.

## Conclusions

The search for novel drug targets against *M. tuberculosis* has been escalated recently under the pressure of the emergence of extensively drug resistant strains [37]. Arylamine N-acetyltransferase is one of the novel targets that plays an important role in cell wall synthesis and intracellular survival of mycobacteria within the macrophage [19]. From a previous HTS [22], the piperidinol scaffold was identified as a selective prokaryotic NAT inhibitor that shows good antimycobacterial activity. In order to explore this scaffold as a possible lead for anti-tubercular therapies, a series of inhibitors was tested for their activity against TBNAT and MMNAT and for their antimycobacterial activity. In addition to inhibiting NAT activity, the compounds were potent against *M. tuberculosis* with an MIC below 17 µM. The data do not preclude the presence of additional targets within *M. tuberculosis*. However, the concept of poly-pharmacy in which one drug has multiple targets is an extremely useful asset in drug design, particularly for antimicrobials where resistance is a major consideration [38].

A novel mechanism of NAT inhibition by the piperidinols was revealed by MS-analysis, and from the 3D-structure of the MMNAT-1 complex. The mechanism of inactivation of NAT involves the formation of PVKs that form an adduct with the active site cysteine. This mechanism was also observed with acyclic Mannich bases considered for the drug design of antimalarial agents [39].

Drug leads that exhibit activation followed by covalent modification of targets have been proposed to be beneficial in developing new TB therapies [27], [40]. This approach has indeed been historically successful with the front-line anti-tubercular drug isoniazid and the related drug ethionamide both retrospectively shown to be prodrugs that require activation to inhibit mycolic acid synthesis. The activated intermediates for those agents form a covalent adduct with the biological molecule NAD [41].

Specific covalent enzyme inactivators have gained recent interest in drug design [42] as being usually associated with lower doses and a longer duration of action, as well as avoiding resistance [42]. The possible toxicity associated with such a mechanism requires the careful design of highly selective agents. It is especially important to improve the stability of these compounds in relation to other cysteine or sulfhydryl bearing entities such as glutathione and mycothiol in mycobacteria. However, as studies on cytotoxicity and the effects on NAT enzymes suggest that these reagents do have inbuilt specificities for certain NAT enzymes, this does not preclude their activity against other mycobacterial proteins.

The piperidinol group presented in this study provides a starting point for novel anti-tubercular agents to add to a growing drug development pipeline in the fight against TB [43], [44].

## Materials and Methods

All chemicals and reagents were purchased from Sigma Aldrich (Poole, Dorset, UK), unless otherwise stated. U937 cells were obtained from the cell bank of the Sir William Dunn School of Pathology, South Parks Road, Oxford [45].

### Range of inhibitors

**Figure 10:**
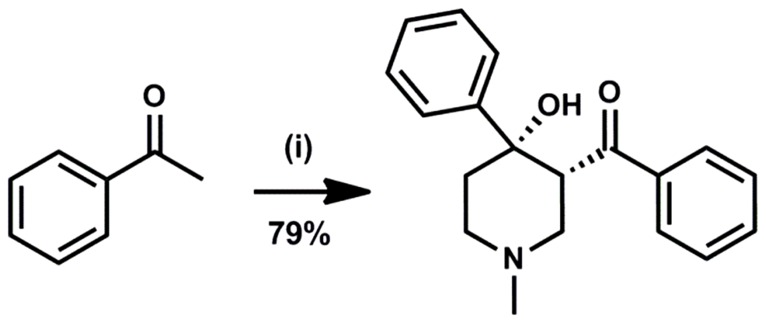
Reagents and conditions: (i) MeNH_2_.HCl, paraformaldehyde, MeCN, cat. HCl, Δ, 16 h.

Compound **1**, which was identified from a previous high-throughput screen was synthesized *ab initio* to confirm its identity and activities as previously described [46]. It has been established previously that during the cyclisation only the diastereoisomer **1** depicted in Figure 10 is formed [46]. The compound was obtained at a 79% yield, with greater than 99.5% purity as determined by reversed phase high performance liquid chromatography (RP-HPLC).

### Preparation of (4-hydroxy-1-methyl-4-phenylpiperidin-3-yl)(phenyl)methanone 1

Compound **1** was prepared as described in Figure 10. Amine hydrochloride (0.25 eq) was added to a stirred solution of aryl methyl ketone (1 eq) and paraformaldehyde (1 eq) in acetonitrile, and the mixture was heated at reflux (82°C) for 20 hours in the presence of a catalytic amount of hydrochloric acid. The reaction mixture was allowed to cool to room temperature, and concentrated *in vacuo*. The resulting solid was dissolved in CH_2_Cl_2_, washed with sat. aq. NaHCO_3_ solution, water and brine, dried, filtered and concentrated *in vacuo*. Following this, acetophenone (2.00 g, 16 mmol), paraformaldehyde (0.49 g, 16 mmol) and methylamine hydrochloride (0.28 g, 4 mmol) were reacted to give a crude product which after purification by column chromatography (diethyl ether, triethyl amine (1%)), furnished **1** (0.93 g, 79%) as a white solid: mp 137–139°C (lit.,^2^ 138–140°C). δ_H_ (400 MHz, CDCl_3_) 1.80–1.88 (1H, m), 2.02–2.19 (1H, m), 2.43 (3H, s), 2.65–2.89 (3H, m), 2.94–2.97 (1H, m), 4.46 (1H, br s), 5.13–5.17 (1H, m), 7.16–7.89 (10H, m).

### Dehydration of compound 1

**Figure 11:**
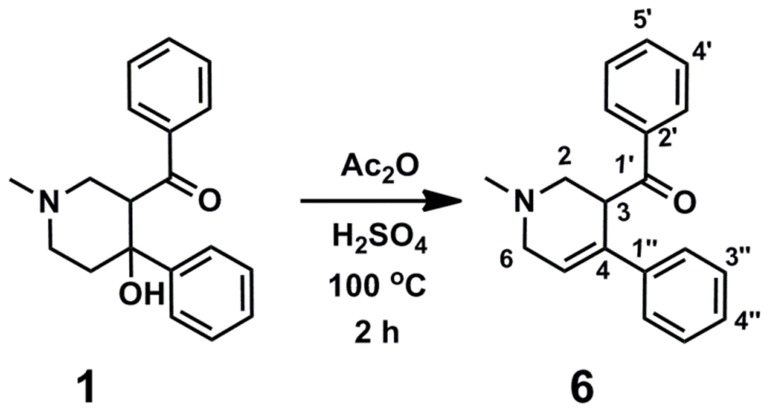
The chemical dehydration of compound 1.

Dehydration of compound **1** in the presence of acetic anhydride was performed as described previously and as shown in Figure 11 [47]. Compound **1** (100 mg, 0.34 mmol) was suspended in acetic anhydride (1.0 mL) and treated with concentrated H_2_SO_4_ (1 drop). The mixture was heated to 100°C for 2 h then carefully added to NaHCO_3_ solution (70 mL sat. aq.). Solid NaHCO_3_ was added until the mixture was made basic and the aqueous phase extracted with ethylacetate (EtOAc) (3×20 mL). The combined organic extracts were dried over MgSO_4_ and concentrated *in vacuo*. The residue was purified by flash chromatography (40–50% EtOAc/Petrol+0.1% triethylamine (Et_3_N)) to give 1-methyl-3-benzoly-4-phenyl-1,2,5,6-tetrahydropyridine (**6**) as an orange oil (67 mg, 71% yield): ν_max_ (neat)/cm^−1^ 2939 (CH), 2785 (CH), 1685 (C = O), 1447, 1027, 693; δ_H_ (400 MHz, CDCl_3_) 2.34 (3H, s, NMe), 2.89 (1H, dd, *J* 11.5, 5.4, 2-*H*H), 2.96 (1H, dd, *J* 11.5, 5.4, 2-H*H*), 3.09 (1H, app. dt, *J* 17.0, 2.9, 6-*H*H), 3.36 (1H, ddd, *J* 17.0, 2.9, 2.1, 6-H*H*), 4.86 (1H, m, 3-H), 6.32 (1H app. td, *J* 2.9, 0.7, 5-H), 7.17 (1H, app. tt, *J* 7.4, 1.4, 4″-H), 7.23 (2H, app. t, *J* 7.4, 3″-H), 7.29 (2H, app. dd, *J* 7.4, 1.4, 2″-H), 7.48 (2H, app. t, *J* 7.5, 4′-H), 7.58 (1H, app. tt, *J* 7.5, 1.5, 5′-H), 8.01 (2H, app. dd, *J* 7.5, 1.5, 5′-H); δ_C_ (75 MHz, CDCl_3_) 45.6 (NMe), 46.9 (C-3), 54.9 (C-6), 55.8 (C-2), 125.3 (C-2″), 125.7 (C-5), 127.0 (C-4″), 128.4 (C-3″), 128.5 (C-3′), 128.7 (C-4′), 133.0 (C-5′), 134.0 (C-4), 136.1 (C-2′), 140.1 (C-1″), 199.0 (C-1′); *m/z* (ESI) 300 ([MNa]^+^ 25), 278 ([MH]^+^, 83).

The phenyl vinyl ketone was synthesised as described previously [48]. The corresponding NMR spectra are shown in the Supporting Information S1.

### Commercially available compounds

Compound **2** was used as a representative of a halide substitution at the benzene ring. To investigate the structural influence of the nitrogen atom functionality over both NAT inhibition and the antimycobacterial activity, two commercially available compounds (**3** and **4**, Cheshire Biosciences, UK) with different substitutions at the piperidinol nitrogen were selected for testing. The synthetic intermediate bis-Mannich base of **1** was also purchased and tested (**5**). The purchased compounds were provided at >95% purity and were used as supplied from the vendors without further characterization or purification. The stock solutions of the test compounds were prepared in dimethyl sulphoxide (DMSO) and stored at −20°C.

Details of source and purity are shown in Table S1.

### Protein production

The NAT enzymes from *M. smegmatis* [49], *S. typhimurium* [21], *P. aeruginosa* [50], *M. marinum* [23], *M. tuberculosis* [51], hamster NAT2 [52] and human NAT1 [53] were produced as recombinant proteins and purified as previously described.

### NAT inhibition assay

An assay for measuring the formation of CoA was used to determine the activity of the enzyme in the presence of potential inhibitors [54]. All the tested compounds were dissolved in dimethylsulphoxide (DMSO) and all reactions were carried out in the presence of 5% (v/v) DMSO. The enzyme (100–150 ng) was mixed with the inhibitors **1–5** (5 µL at a final concentration of 0–250 µM) and incubated for 15 min at 24°C prior to starting the reaction by adding 15 µL hydralazine and 12 µL Ac-CoA at final concentrations of 150 µM and 120 µM respectively, in a final total volume of 100 µL of 20 mM Tris-HCl pH 8. The assay was performed as an end-point read out measurement by stopping the reaction after 10 min at 24°C using 25 µL Ellman's reagent (5 mM 5,5′-dithiobis-(2-nitrobenzoate) solution in 6.4 M guanidine-HCl and 100 mM Tris-HCl, pH 7.3). The absorbance was measured at a wavelength of 405 nm within 2 min (Tecan Sunrise Plate Reader). The assays were repeated using a 10-fold enzyme concentration to exclude promiscuous non-specific inhibitors [55]. The activity of the enzyme in the presence of 5% (v/v) DMSO was measured as a control. Inhibition values were determined as the ratio of the enzyme activity (expressed as the rate of CoA formation per microgram protein (µM/min.µg)) with the requisite compound, to the activity of the control without inhibitor. IC_50_ values were determined from the inhibition curves which were obtained by non-linear fitting of the % inhibition and the inhibitor concentration (µM) using the Log(inhibitor) vs. response module of GraphPad Prism 5.0.

For the reversibility studies, excess inhibitor was measured by dialysis. Each enzyme (0.07 mM MMNAT or TBNAT in 20 mM Tris-HCl pH 8 and 5% (v/v) DMSO) was preincubated either alone or with 15-fold molar excess of compound **1** in a final volume of 50 µL at 24°C for 1 h. Each sample was then dialysed against 1 L fresh assay buffer (20 mM Tris-HCl pH 8) at 4°C for 16 h. The enzyme activities of the samples were measured before dialysis and then measured after dialysis by measuring the rate of Ac-CoA (120 µM) hydrolysis in the presence of 150 µM HLZ.

For the determination of the time dependence of interaction between enzymes and inhibitors, the inhibitor was diluted to less than 1% concentration. Incubation mixtures (20 µL) contained 0.06 mM MMNAT or TBNAT in 20 mM Tris-HCl, pH 8 and 5% (v/v) DMSO and variable concentrations of the inhibitors (0–50 µM) were prepared using the protocol described in Figure S1. Aliquots of 1 µL were removed from the reaction mixture at different time points (incubation-time) and diluted to 100 µL using the assay buffer containing HLZ and Ac-CoA to the final concentrations of 150 µM and 120 µM, respectively. The rate of Ac-CoA hydrolysis was measured over a three-minute time period. The reaction was stopped with 25 µL Ellman's reagent in 6.4 M guanidine and the absorbance was measured at a wavelength of 405 nm. The residual activity was measured as a percentage of a control prepared as described in Figure S1 and plotted against the incubation-time. The data were fitted using the Semilog line (X is linear, Y is Log) module of GraphPad Prism 5.0. The slope of each line is equivalent to (−k_obs_/2.303) at each inhibitor concentration.The controls in which no enzyme was present using CoA and Ellman's reagent gave the same results whether compound **1** was present or not.

### Mycobacterial growth inhibition *in vitro*

Mycobacteria (*M. bovis* BCG and *M. tuberculosis* H37Rv) were grown as spot cultures in 6-well plates on solid medium (Middlebrook 7H10 medium supplemented with 10% (v/v) oleic acid-albumin-dextrosecatalase (OADC)) as previously described [19], with test compounds at the concentrations indicated in the text. Test compounds were added to the melted, partially cooled 7H10-OADC agar medium as solutions in DMSO, and the final concentration of DMSO in each well was 0.1% (v/v). The MIC is defined as the concentration of an inhibitor at which no growth of mycobacteria was detected after a period of 2 weeks.

### Cytotoxicity

The mouse macrophage cell-line RAW 264.7 (ATCC no. TIB 71) were grown as a monolayer for 48 h in RPMI 1640-FBS complete medium either in presence of 0.1% (v/v) DMSO alone or in presence of the piperidinol derivative as well as its four analogues (**1–5**) dissolved in 0.1% (v/v) DMSO or isoniazid as control. The percentage viable RAW cells was determined following detachment of the cells with lidocaine/EDTA as described previously [56] by Trypan blue exclusion. The percentage viability was determined in triplicate from counting at least 200 cells per field. as described previously [56]. Human U937 [45] cells were also tested with compounds following growth in suspension culture as described previously [45].

### Electrospary ionization mass spectrometry (ESI-MS)

ESI-MS was performed as described previously [57]. A sample of 2.5 mg/mL (80 µM) MMNAT was analyzed on a Micromass LCT mass spectrometer. The samples were pretreated with DMSO or 0.08–1.2 mM inhibitor dissolved in DMSO to a final percentage of 5% (v/v) DMSO and incubated for 30–60 min before analysis. ESI-MS analysis was performed in positive ion mode after denaturation in 50% v/v acetonitrile in water with an accuracy of ±0.1%. The sample was run over a desalting column prior to MS analysis.

### Liquid chromatography-mass spectrometry (LC/MS)

For the LC/MS measurements, samples of cysteine, compound **1**, and a 1∶1-mixture of both were used in 100 µM solutions in MOPS buffer pH 8.0. After 16 h incubation, all samples were mixed with 5 µL of the primary amine derivatization reagent AccQ-Tag Ultra, Waters® (6-aminoquinolyl-N-hydroxysuccinimidyl carbamate) and injected onto a reverse phase Acquity C18 column (2.1×100 mm, 1.7 µm particles) equilibrated with 5% AccQ Tag Ultra Eluent A on an Acquity Ultra Performance Liquid Chromatography system. Derivitized substrate and products were resolved using a gradient of increasing AccQ Tag Ultra Eluent B solution, detected by absorbance at 260 nm [58].

### Co-crystallisation of MMNAT-1 complex

Protein crystallisation was performed using the sitting- drop vapour-diffusion technique. Sitting crystallisation drops were set up in 96-well plates containing commercially available sparse matrix and systematic grid screen conditions.

For co-crystallisation of MMNAT with compound **1**, the protein (10 mg/mL) was incubated with 5 mM compound **1** in 20 mM Tris-HCl pH 8.0 containing 5% (v/v) DMSO for 1 h at 24°C. The protein was buffer exchanged with fresh Tris-HCl buffer (20 mM Tris-HCl pH 8.0) to remove the excess inhibitor and re-concentrated to 10 mg/mL. Initial high-throughput screens to identify crystallization conditions were performed using a Tecan Genesis Pro Team 150 Robot (Tecan). Equal volumes (100 nL) of mother-liquor and protein were set up as sitting drops using a Mosquito crystallisation robot (TTP Labtech). A preliminary screen for suitable crystallisation conditions at 19°C was carried out using the JCSG-plus, PACT and Morpheus sparse-matrix screens (288 conditions). Crystals of the MMNAT-POP complex grew in condition E2 of the JCSG-plus screen (0.2 M NaCl, 0.1 M Na-cacodylate pH 6.5 and 2.0 M (NH_4_)_2_SO_4_). For cryo-protection, crystals were briefly (10–30 s) washed with a 7 M sodium formate solution, and were then flash cryo-cooled into liquid nitrogen.

Native data were collected at 100 K at Diamond Light Source beamline I04. The data were integrated and scaled using XDS [59] and SCALA [60] within the CCP4 program suite (Collaboration Computational Project, Number 4, 2011) [61]. The crystal structure was solved by molecular replacement (MR) using the program PHASER MR [62] using a previously determined native MMNAT crystal structure, stripped of heteroatoms, as a search model (PDB code: 3LTW, 2.1 Å). Rigid body refinement of the MR solution and the remaining cycles of restrained refinement were carried out with REFMAC5 [63] and autoBUSTER [64]. Molecular models of the substrate were constructed using Grade [64], while model building was performed using COOT [65]. The stereochemical properties and quality of the final model were assessed with the program MOLPROBITY [66]. Structural figures and graphical renderings were made with either PYMOL [67] or Discovery Studio (DS) Visualizer 3.1 [68].

The coordinates have been deposited in the Protein Data Bank, disposition code 4B55.

## Supporting Information

Figure S1 **The time-dependent inhibition of TBNAT and MMNAT by compound 1.**
(TIF)

Figure S2 **Flow chart outlining both the substrate-protection protocol and the time-dependent inhibition protocol.** Measurements were performed using 0.06 mM MMNAT or TBNAT in buffer A. [E] represents the enzyme concentration required for initial linear kinetics. [I] is the stated inhibitor concentration. Buffer A consisted of 20 mM Tris-HCl, pH 8 and 5% DMSO.
(TIF)

Supporting Information S1 **NMR spectra for compounds 1, 6 and PVK.**
(PDF)

Table S1 **The chemical structure of compound 1 and its analogues.**
(DOCX)

Table S2 **Data collection, processing and refinement statistics for the MMNAT-POP complex structure determination.**
(DOCX)
