# Supplementary figures and images for: Piperidinols That Show Anti-Tubercular Activity as Inhibitors of Arylamine N-Acetyltransferase: An Essential Enzyme for Mycobacterial Survival Inside Macrophages
Source: PLoS One. 2012 Dec 28;7(12):e52790. doi: 10.1371/journal.pone.0052790 (PMC3532304; doi:10.1371/journal.pone.0052790)

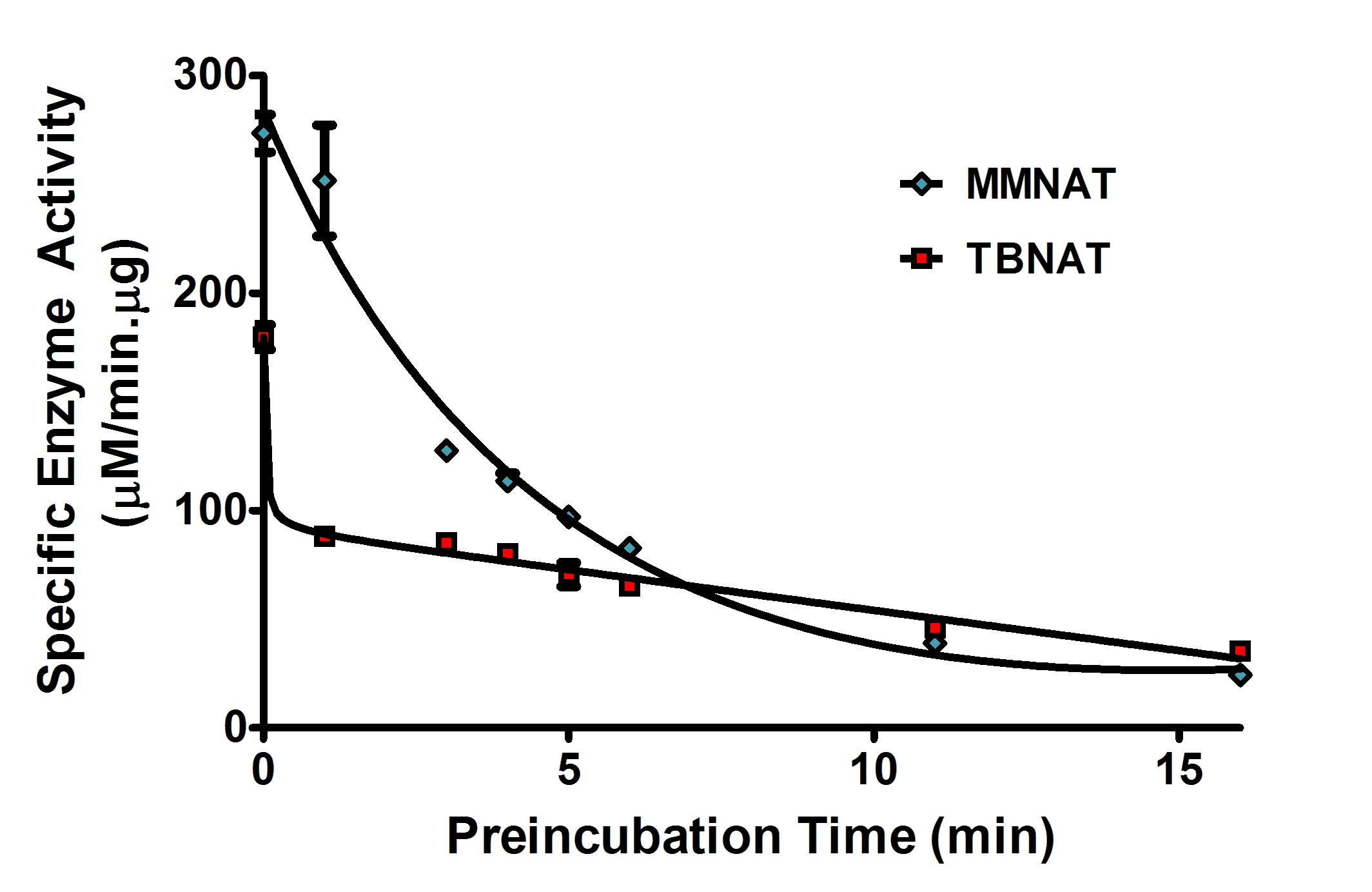

Supplement: Figure S1 — The time-dependent inhibition of TBNAT and MMNAT by compound 1. (TIF) [file pone.0052790.s001.tif]
