# Supplementary material for: Piperidinols That Show Anti-Tubercular Activity as Inhibitors of Arylamine N-Acetyltransferase: An Essential Enzyme for Mycobacterial Survival Inside Macrophages
Source: PLoS One. 2012 Dec 28;7(12):e52790. doi: 10.1371/journal.pone.0052790 (PMC3532304; doi:10.1371/journal.pone.0052790)

Compound **1** ^{1}H NMR: 400 MHz $CDCl_3$ Bruker AV400 spectrometer

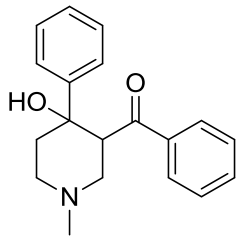

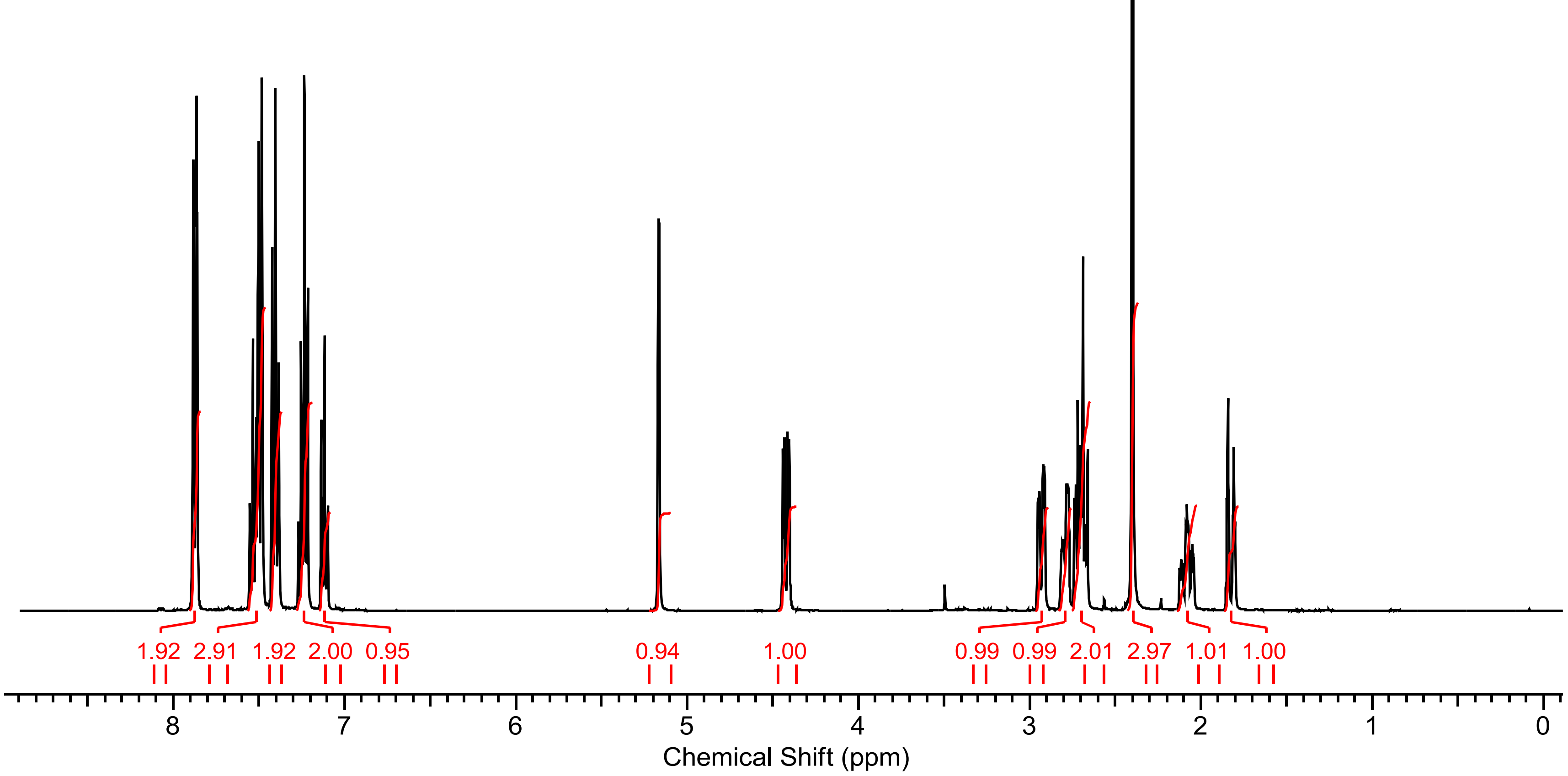

Compound **1** ^{13}C NMR: 75 MHz $CDCl_3$ Bruker DPX300 spectrometer

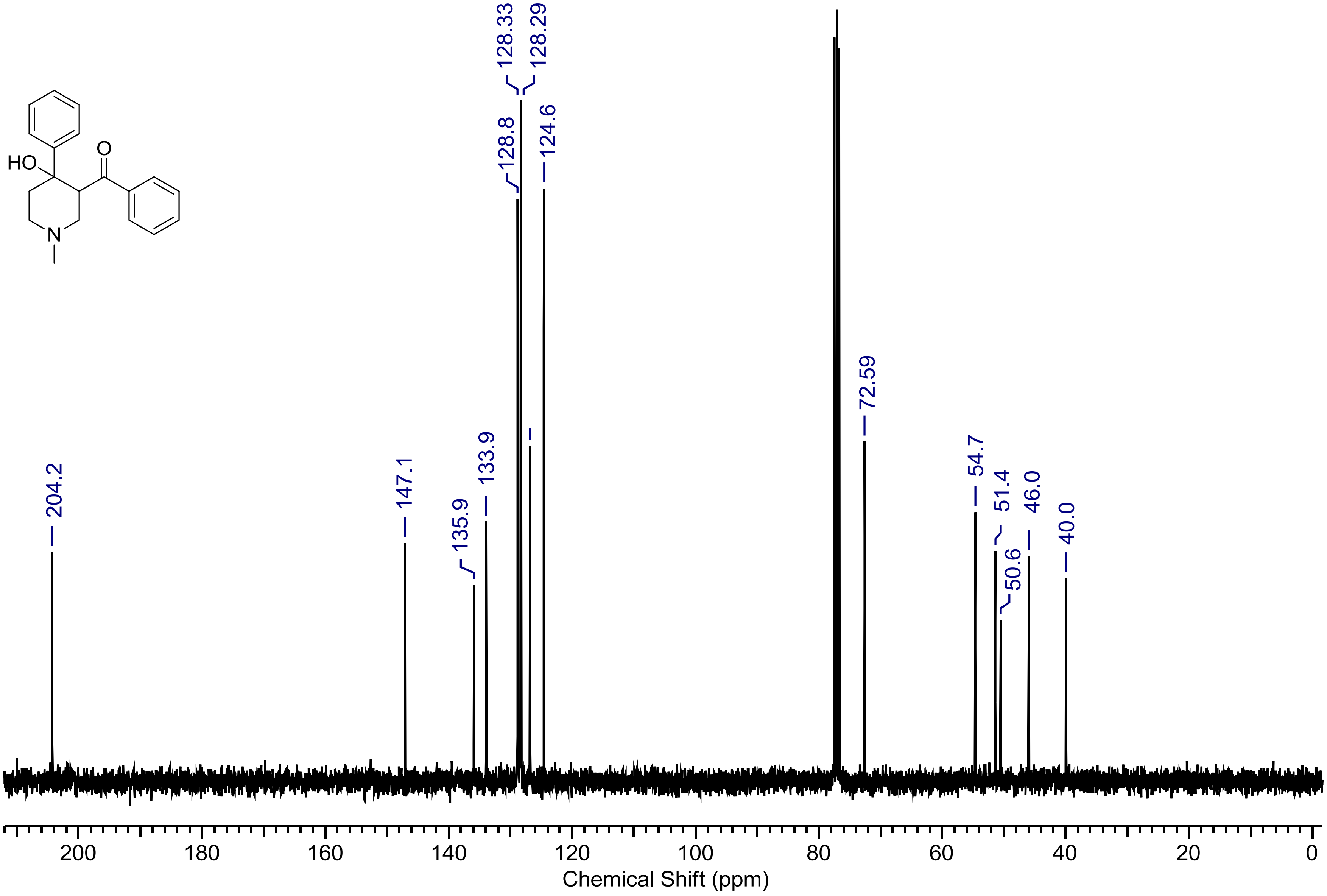

Compound **6** ^{1}H NMR: 400 MHz $CDCl_3$ Bruker AV400 spectrometer

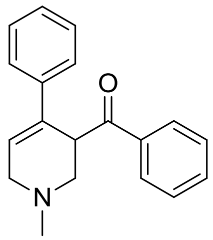

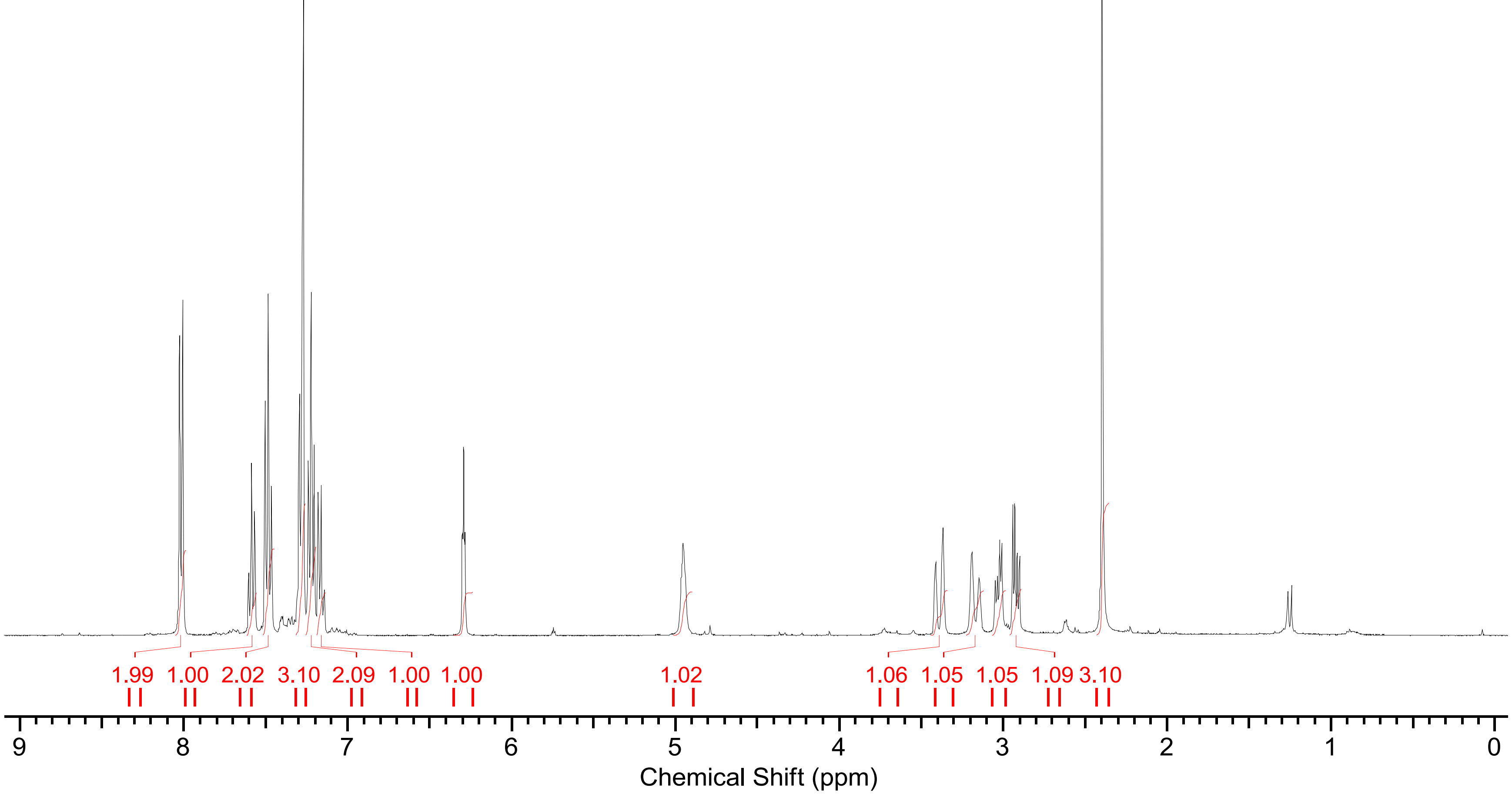

Compound **6** ^{13}C NMR: 75 MHz $CDCl_3$ Bruker DPX300 spectrometer

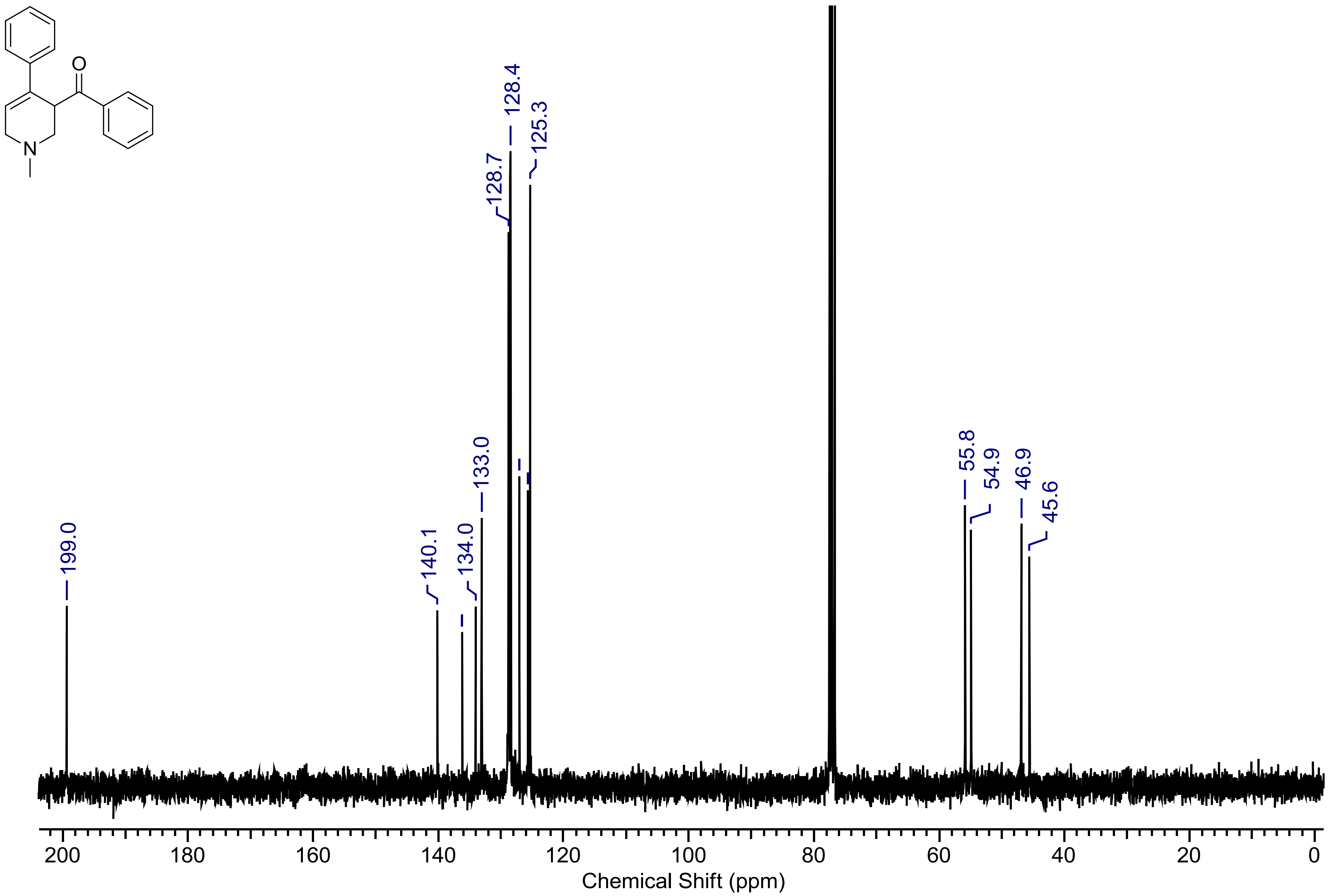

Phenyl vinyl ketone ^{1}H NMR: 400 MHz $CDCl_3$ Bruker AV400 spectrometer

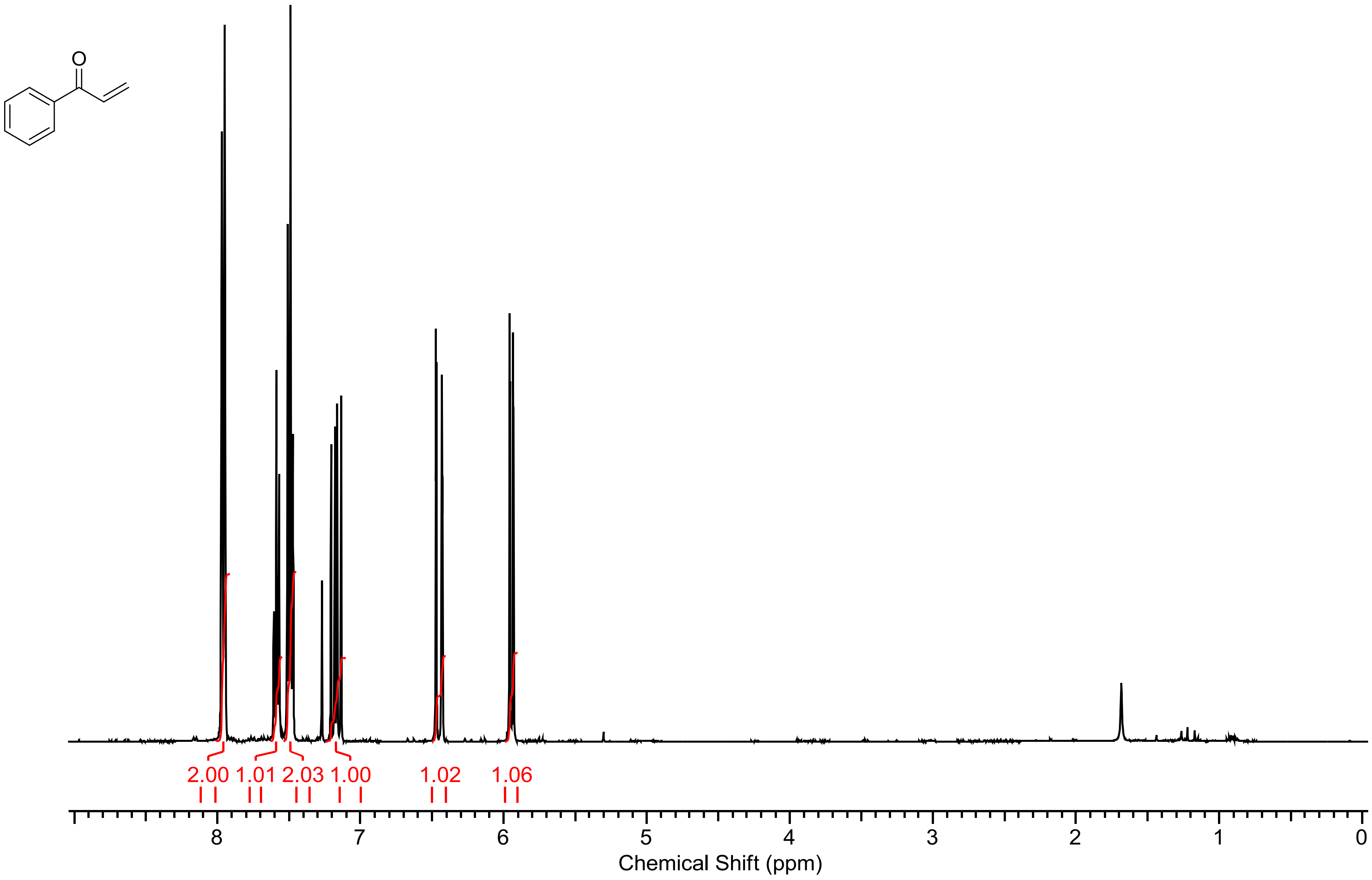

Phenyl vinyl ketone ^{13}C NMR: 100 MHz $CDCl_3$ Bruker AV400 spectrometer

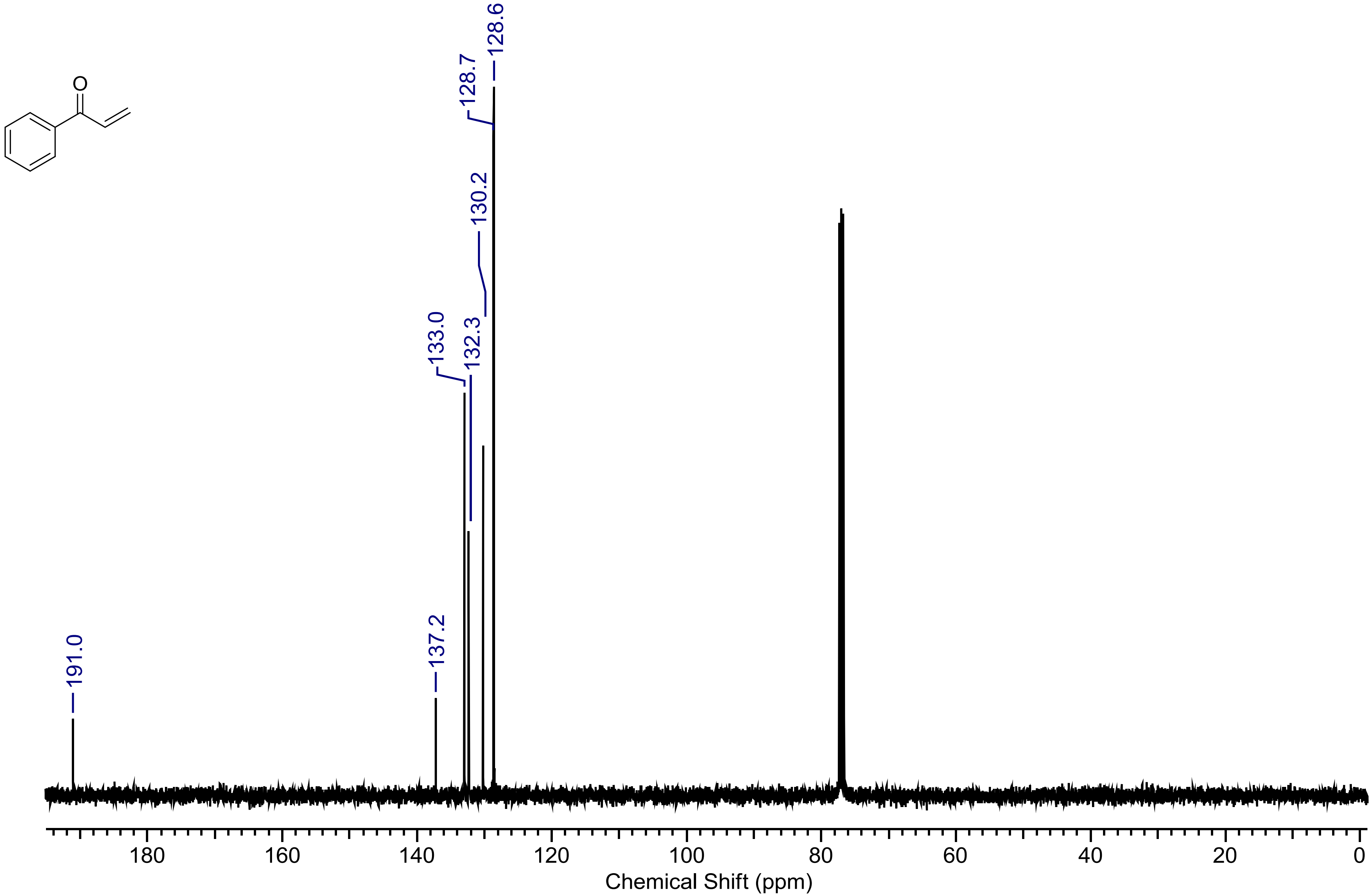

Supplement: Supporting Information S1 — NMR spectra for compounds 1, 6 and PVK. (PDF) [file pone.0052790.s003.pdf]
