# Supplementary material for: Piperidinols That Show Anti-Tubercular Activity as Inhibitors of Arylamine N-Acetyltransferase: An Essential Enzyme for Mycobacterial Survival Inside Macrophages
Source: PLoS One. 2012 Dec 28;7(12):e52790. doi: 10.1371/journal.pone.0052790 (PMC3532304; doi:10.1371/journal.pone.0052790)
Supplement: Table S1 — The chemical structure of compound 1 and its analogues. (DOCX) [file pone.0052790.s004.docx]

**Table S1: The chemical structure of compound 1 and its analogues.**

| **Code** | **R1** | **R2** | **Source** |  | **Purity** |
| --- | --- | --- | --- | --- | --- |
| **1** | H | -CH_3_ | Synthesised |  | > 95 % |
| **2** | Cl | -CH_3_ | ChemDiv |  | > 95 % |
| **3** | H |  | Cheshire Biosciences |  | > 95 % |
| **4** | H |  | Cheshire Biosciences |  | > 95 % |
| **5** |  | | Sigma |  | > 95 % |
| **6** |  | | Synthesised |  | > 95 % |
