# Supplementary material for: Piperidinols That Show Anti-Tubercular Activity as Inhibitors of Arylamine N-Acetyltransferase: An Essential Enzyme for Mycobacterial Survival Inside Macrophages
Source: PLoS One. 2012 Dec 28;7(12):e52790. doi: 10.1371/journal.pone.0052790 (PMC3532304; doi:10.1371/journal.pone.0052790)
Supplement: Table S2 — Data collection, processing and refinement statistics for the MMNAT-POP complex structure determination. (DOCX) [file pone.0052790.s005.docx]

**Table S2: Data collection, processing and refinement statistics for the MMNAT-POP complex structure determination. ^a,b^**

|  |  |  | **MMNAT-POP** |  |
| --- | --- | --- | --- | --- |
| **Data collection** |  |  |  |  |
| **Beamline** |  |  | Diamond I04 |  |
| **Detector** |  |  | ADSC Q315r CCD |  |
| **λ (Å)** |  |  | 0.976 |  |
| **Temperature (K)** |  | 100 | | |
| **Space group** |  | P 4_1_ 2_1_ 2 | | |
| **Unit cell dimensions:** |  |  | 51.84, 176.75 |  |
| **a = b, c (Å)** |  |  |  |  |
| **α = β = γ (º)** |  | 90 | | |
| **Resolution range (Å)** |  |  | 19.5- 2.7 (2.8-2.7) |  |
| **Measured reflections** |  |  | 49487 (4473) |  |
| **Unique reflections** |  |  | 7020 (875) |  |
| **Multiplicity** |  |  | 7.0 (5.1) |  |
| **Overall I/σ(I)** |  |  | 15.5 (3.0) |  |
| **Overall completeness (%)** |  |  | 96.0 (87.9) |  |
| **Overall R_merge_** |  |  | 0.097 (0.5) |  |
| **R_meas_** |  |  | 0.11 (0.56) |  |
| **R_pim_** |  |  | 0.038 (0.23) |  |
| **Solvent content (%)** |  |  | 37.9 |  |
|  |  |  |  |  |
| **Refinement** |  |  |  |  |
| **R_cryst_ (Fo>4sFo; Fo)** |  |  | 0.23 |  |
| **R_free_ (Fo>4sFo; Fo)** |  |  | 0.29 |  |
| **Refined residues** |  |  | 271 |  |
| **Refined water molecules** |  |  | 39 |  |
| **Refined inhibitor molecules** |  |  | 1 |  |
| **R.M.S.D for bond lengths (Å)** |  |  | 0.005 |  |
| **R.M.S.D for bond angle (º)** |  |  | 0.82 |  |
| **Ramachandran plot** |  |  |  |  |
| **Most favoured regions (%)** |  |  | 95.9 |  |
| **Allowed regions (%)** |  |  | 3.3 |  |
| **Disallowed region (%)** |  |  | 0.74 |  |

^a^ The MMNAT-POP crystal was obtained by co-crystallisation of MMNAT with compound **1**. ^b^ Values in parentheses are the statistics for the highest resolution shell. PDB code 4B55.
